# An introduction to Sequential Monte Carlo for Bayesian inference and model comparison—with examples for psychology and behavioral science

**DOI:** 10.3758/s13428-025-02642-1

**Published:** 2025-03-26

**Authors:** Max Hinne

**Affiliations:** Donders Institute for Brain, Cognition, and Behaviour, Radboud University, Nijmegen, The Netherlands

**Keywords:** Bayesian inference, Sequential Monte Carlo, Bayesian model comparison, Marginal likelihood, Computational modelling

## Abstract

Bayesian inference is becoming an increasingly popular framework for statistics in the behavioral sciences. However, its application is hampered by its computational intractability – almost all Bayesian analyses require a form of approximation. While some of these approximate inference algorithms, such as Markov chain Monte Carlo (MCMC), have become well known throughout the literature, other approaches exist that are not as widespread. Here, we provide an introduction to another family of approximate inference techniques known as *Sequential Monte Carlo* (SMC). We show that SMC brings a number of benefits, which we illustrate in three different examples: linear regression and variable selection for depression, growth curve mixture modeling of grade point averages, and in computational modeling of the Iowa Gambling Task. These use cases demonstrate that SMC is efficient in exploring posterior distributions, reaching similar predictive performance as state-of-the-art MCMC approaches in less wall-clock time. Moreover, they show that SMC is effective in dealing with multi-modal distributions, and that SMC not only approximates the posterior distribution but simultaneously provides a useful estimate of the marginal likelihood, which is the essential quantity in Bayesian model comparison. All of this comes at no additional effort from the end user.

## Introduction

Bayesian inference forms a powerful and elegant framework for learning about causes from observed effects (Gelman et al., 2013), and is becoming increasingly popular as a computational modeling tool within behavioral science (Heck et al., 2023; Schad et al., 2021; Van De Schoot et al., 2017; Andrews & Baguley, 2013). While the expressive capabilities of the Bayesian approach are almost limitless, in practice it does suffer from one major drawback: Bayesian inference is computationally intractable, as the normalization of the posterior distribution usually consists of a high-dimensional integral without closed-form solution for nearly all realistically relevant models. Fortunately, not all is lost, as great achievements have been made in the realm of *approximate inference*. General techniques such as Markov chain Monte Carlo (MCMC) and variational inference (VI) are widely used and still actively being developed, which ensures that many Bayesian models can be applied in practice, despite their intractability. However, that is not to say that no difficulties remain. For example, many of the existing approximate inference techniques have trouble with multimodal distributions, where distinct parameter configurations have the same probability. Furthermore, estimating the marginal likelihood of a Bayesian model (which is essential for model comparison and averaging) is notoriously challenging.

In this paper, we provide a tutorial on Sequential Monte Carlo (SMC), another framework for approximate Bayesian inference, which addresses several of these challenges. Although the algorithm will later be discussed in detail, we provide a simple intuition here. First, consider the ‘standard’ MCMC approach. Here, the procedure consists of a guided random walk through the landscape of possible values of the parameter of interest. The random walk is constructed in such a way that the time spent at any location is proportional to its (desired) posterior probability. In SMC, rather than performing a single, lengthy, random walk, we perform a large number of short explorations in parallel. After their short excursions, we evaluate the locations that the walks have ended up in. From those walks that ended up in a desired location (which are those with a relatively high likelihood), new walks are initiated, while the unsuccessful tours are terminated. After a number of iterations of short parallel walks have been performed, the end locations of all remaining walks are aggregated as the approximate posterior. To put it bluntly, the SMC algorithm replaces serial exploration of the posterior by parallel exploration. With the right computer hardware, the parallel approach can be much more time efficient. The intricacies of the SMC algorithm reside in the way the random walks are compared and combined, and how they are guided towards the desired posterior. These steps are explained in detail throughout this introduction.

It is important to emphasize that there are two distinct modes in which SMC can be applied. Originally, SMC was developed for the estimation of time series models, in which data are obtained sequentially (Kantas et al., 2009). Here, the different explorations of the SMC algorithm represent the temporal evolution of a system, such as the position of a vehicle (for a tutorial on this view on SMC, we refer the reader to Speekenbrink (2016)). More recently, however, we witness a shift to another perspective on SMC. With the development of advanced variants of SMC (which we will discuss later) (Fearnhead & Taylor, 2013; Mlikota & Schorfheide, 2023) and the increasing availability of parallel compute hardware (Lee et al., 2010), it has become appealing to use SMC for approximate Bayesian inference for ‘static’ models (Chopin, 2002), as an alternative to Markov chain Monte Carlo (Wills & Schön, 2023; Speich et al., 2021; Gunawan et al., 2020). In this view, all data are assumed to be present from the onset of the inference, and the different explorations of the SMC algorithm represent potential values for latent variables of a model. It is this view on SMC that we discuss in this paper. We focus on how SMC can be seen as an alternative to MCMC, as well as on the implications of using MCMC as a component within the larger SMC algorithm. In contrast to MCMC, SMC tends to provide better representations of multimodality in our target distributions. Moreover, although the name suggests otherwise, within SMC many of the actual computations can be performed in parallel, which can be a tremendous advantage in the day and age of parallel compute hardware (Lee et al., 2010). A final but important advantage of SMC is that it provides an estimate of the marginal likelihood (Chopin & Papaspiliopoulos, 2020) which can be used for Bayesian hypothesis testing. This eliminates the need for additional analyses, such as importance sampling or bridge sampling.

With this tutorial, we aim to make SMC accessible to researchers in the field of (computational) psychology. To do so, we first provide the theoretical foundation of the algorithm, and then discuss in detail three example problems that showcase the strengths of SMC. The paper is structured as follows. In Section “The challenge of Bayesian inference”, we outline the essentials of (approximate) Bayesian inference and indicate where the computational challenge comes from. In Section “Markov chain Monte Carlo approximations of the posterior”, the Metropolis–Hastings MCMC algorithm is discussed briefly. This is an essential prerequisite, as it is part of the larger SMC algorithm, which we describe in detail in Section “Sequential Monte Carlo approximations of the posterior and the marginal likelihood”. Here, we also describe two pragmatic extensions to the core algorithm, namely *adaptation* and *tempering*. We also show how one can estimate the marginal likelihood of a model with SMC at (nearly) no additional computation. In the second part of the paper, we provide examples that focus both on estimation of the posterior as well as the marginal likelihood, and we demonstrate how SMC is a competitive choice for both. Finally, in Section “Discussion”, we discuss the potential as well as the limitations of SMC for statistical modeling.

To accompany the paper, we provide several code examples using the Python Jax framework (Bradbury et al., 2018), in particular with the libraries (Hinne, 2025) for modeling, Blackjax (Cabezas et al., 2023) for MCMC/SMC sampling and Distrax (DeepMind et al., 2020) for probability distributions. Code for these worked examples is available on GitHub.

## The challenge of Bayesian inference

Bayesian modeling typically proceeds as follows: we observe a set of variables, *D*, but we are interested in (the distribution over) their causes, the latent variables $\theta$. Following Bayes’ theorem, we know that the distribution of $\theta$ conditioned on the observations is given by

1 $$\begin{aligned} \overbrace{p(\theta \mid D)}^{\text {posterior}}&= \frac{\overbrace{p(D\mid \theta )}^{\text {likelihood}} \overbrace{p(\theta )}^{\text {prior}}}{\underbrace{p(D)}_{\textrm {marginal likelihood}}} \\&= \frac{p(D\mid \theta )p(\theta )}{\int p(D\mid \theta )p(\theta ) \, \text {d}\theta } .  \end{aligned}$$

In this fundamental expression, the prior distribution $p(\theta )$ represents our beliefs about the latent variables $\theta$ before any observations have been made. The likelihood $p(D\mid \theta )$ describes what, given a cause $\theta$, the distribution over the observations *D* would be. Following basic axioms of probability theory, the equation then tells us how we obtain the posterior distribution $p(\theta \mid D)$, representing our updated beliefs. In most situations, this distribution is what we need to answer our research questions, but in other times we are interested in the *marginal likelihood* $p(D) = \int p(D\mid \theta )p(\theta ) \,\text {d}\theta$. This term quantifies the evidence that our observations *D* provide for our model. The relative amount of this evidence between two models, that is, the marginal likelihood of one model divided by that of an alternative model, is a quantity known as the *Bayes factor*. The Bayes factor is the key ingredient in Bayesian model comparison, which makes correctly computing marginal likelihoods essential.

Although Eq. (1) looks fairly harmless, the difficulty of Bayesian inference hides in the integral in the marginal likelihood. Except for very specific cases^1^, this integral (and hence the posterior) cannot be computed exactly, which makes computing either the posterior or the marginal likelihood (or both) computationally intractable. The way forward is to use *approximations* instead. When we are interested in posterior inference, the most common approximation framework is that of Markov chain Monte Carlo (MCMC). Since MCMC is an essential subroutine of the Sequential Monte Carlo method, we provide a brief introduction to MCMC below. Readers familiar with these techniques and anxious to learn about Sequential Monte Carlo, may skip ahead to Section “Sequential Monte Carlo approximations of the posterior and the marginal likelihood”.

### Markov chain Monte Carlo approximations of the posterior

**Algorithm 1:**
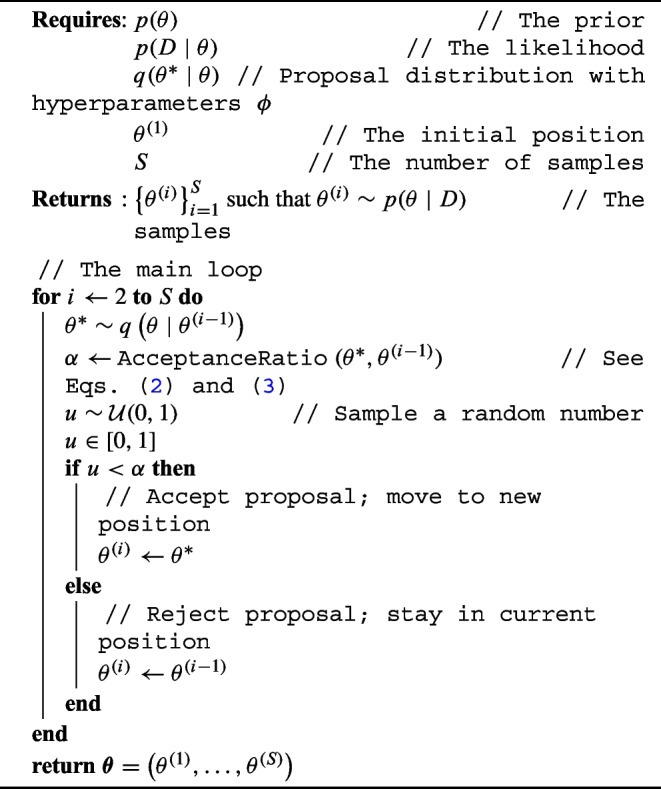
Pseudo-code for the Metropolis–Hastings Markov chain Monte Carlo algorithm.

The key idea of Markov chain Monte Carlo is to construct a random walk on the support of the latent parameters $\theta$, but in a clever way so that the amount of time spend at any location is proportional to the probability of that value. Although many (more advanced) MCMC algorithms exist, for this exposition we focus on the canonical Metropolis–Hastings algorithm, which nicely captures the main ideas of MCMC. It works as follows:

1. Determine a random starting position $\theta ^{(1)}$. For instance, we might draw this value from the prior: $\theta ^{(1)}\sim p(\theta )$. Note that the superscripts in parentheses, such as in $\theta ^{(i)}$, indicate an index, not a mathematical exponentiation.
2. At every subsequent iteration *i* a new position is sampled from a user-defined *proposal distribution* $q\left( \theta ^* \mid \theta ^{(i-1)}\right)$. A common choice for *q* is a Gaussian distribution centered at the current position, which means that a newly proposed position is obtained via $\theta ^* \sim \mathcal {N}\left( \theta ^{(i-1)}, \sigma ^2\right)$. The variance of this distribution $\sigma ^2$ determines how distant from the current position our proposals tend to be.
3. We then decide to *accept* or *reject* this newly proposed value $\theta ^*$. We accept it as the new sample $\theta ^{(i)}$ with probability
  2 $$\begin{aligned} \alpha  &= \text {min}\left( 1, \frac{p\left( \theta ^* \mid D\right) }{p\left( \theta ^{(i-1)} \mid D\right) } \frac{ q\left( \theta ^{(i-1)} \mid \theta ^*\right) }{ q\left( \theta ^*\mid \theta ^{(i-1)}\right) } \right) \\ & = \text {min}\left( 1, \frac{p\left( D\mid \theta ^*\right) }{p\left( D \mid \theta ^{(i-1)} \right) } \frac{p\left( \theta ^*\right) }{p\left( \theta ^{(i-1)}\right) } \frac{q\left( \theta ^{(i-1)} \mid \theta ^*\right) }{ q\left( \theta ^*\mid \theta ^{(i-1)}\right) } \right) . \end{aligned}$$
  If we indeed accept, we set $\theta ^{(i)}=\theta ^*$, else we reject it and set $\theta ^{(i)}=\theta ^{(i-1)}$ (that is, we remain in the old position).
4. Return to step 2 until sufficient samples have been obtained.

The acceptance ratio in Eq. (2) consists of the posterior probability of the proposal, divided by the posterior probability of the current position, corrected for the potential bias as a result of the proposal density via $q\left( \theta ^{(i-1)} \mid \theta ^* \right) / q\left( \theta ^* \mid \theta ^{(i-1)}\right)$, a term known as the Hastings factor. Of course, we don’t actually have access to these posterior probabilities; the whole goal of MCMC is to approximate them, which is intractable due to the normalization constant. This term fortunately drops out in the ratio in Eq. (2) so only the prior, likelihood, and proposal densities must be computed, which are generally (chosen to be) straightforward and tractable. Furthermore, if the proposal distribution is symmetric, then $q\left( \theta ^{(i-1)} \mid \theta ^* \right) = q\left( \theta ^* \mid \theta ^{(i-1)}\right)$, so the Hastings-factor drops out as well, which when taken together results in:

3 $$\begin{aligned} \alpha = \text {min}\left( 1, \frac{p\left( D\mid \theta ^*\right) p\left( \theta ^*\right) }{p\left( D \mid \theta ^{(i-1)} \right) p\left( \theta ^{(i-1)}\right) } \right) . \end{aligned}$$

This equation expresses that the probability of accepting a newly proposed value is proportional to its probability density: a proposal with a higher density is always accepted, whereas a proposal with a lower density is only accepted sometimes, proportional to how much less likely it is. The complete algorithm is shown in pseudo-code in Algorithm 1.

The distribution of sampled $\theta$ is guaranteed to converge to the true posterior $p(\theta \mid y)$ for $S\rightarrow \infty$, where *S* is the number of samples collected in this fashion, but of course, in practice, only finite approximations can be attained. It therefore remains important to assess whether the MCMC approximation has *converged*. Intuitively speaking, from this point on, the samples are no longer determined by their arbitrary initial conditions, but indeed follow the target distribution. We return to this topic in Section “Evaluating the posterior”.

## Sequential Monte Carlo approximations of the posterior and the marginal likelihood

The Metropolis–Hastings MCMC algorithm is easy to implement and has become a staple algorithm for approximate Bayesian inference. In practice, however, it can suffer from different drawbacks, such as requiring a long time to converge, or having trouble accurately exploring distributions with multiple regions of high probability density. The SMC algorithm tends to be more robust against these challenges. At its core, Sequential Monte Carlo (SMC) is another class of approximate inference methods. Originally, it was developed to perform inference in state-space models (Speekenbrink, 2016). In that context, data form a time series, and the model parameters need to be updated sequentially as observations from these time series come in. However, SMC can also be used to perform inference with a static set of observations (Gunawan et al., 2020; Chopin & Papaspiliopoulos, 2020), and for this tutorial we focus on the latter approach.

Just as with MCMC, the specific implementation details can vary considerably, but the general outline is as follows (Fearnhead & Taylor, 2013; Mlikota & Schorfheide, 2023; Speich et al., 2021). Roughly speaking, one can consider the SMC algorithm as the parallel execution of short MCMC chains, known as particles. In each iteration of the algorithm, these particles are resampled so that those corresponding to relatively high likelihood values are continued in the next iteration, whereas the particles with relatively poor scores are terminated. At the final state of the algorithm, the weighted collection of all particles is aggregated as a discrete approximation of the desired distribution.

### The SMC algorithm

More formally, we have at every SMC iteration $t=1, \ldots , T$ a collection of *M* *particles* $\theta _t^{(1)}, \ldots , \theta _t^{(M)}$, as well as a set of corresponding *weights*: $w_t^{(1)}, \ldots , w_t^{(M)}$. The weights are typically initialized as $w_0^{(i)}=1/M, i \in 1, \ldots , M$, and the first value of each particle is drawn from the prior, that is $\theta _1^{(i)} \sim p(\theta )$. Then the iterative procedure starts, which is reminiscent of evolutionary algorithms (Braak, 2006; Vrugt et al., 2009). In each iteration, *t*, a couple of steps take place:

1. First, the fitness of the particles is determined by *re-weighing* them, using the ratio of likelihoods
  4 $$\begin{aligned} w_t^{(i)} = \frac{p_t\left( D \mid \theta _{t-1}^{(i)}\right) }{p_{t-1}\left( D \mid \theta _{t-1}^{(i)}\right) } . \end{aligned}$$
  The subscript *t* in the likelihoods indicates that these densities may be dependent on the SMC iteration. We return to this in the section on adaptive tempering below. Intuitively, the weights reflect how much each particle’s fit to the observations was increased between two successive iterations. The weights are subsequently re-normalized so that they sum to one:^2^
  5 $$\begin{aligned} \tilde{w}_t^{(i)} = \frac{w_t^{(i)}}{\sum _{j=1}^M w_t^{(j)}} . \end{aligned}$$
  This step ensures that the particles are weighted proportional to their likelihood.
2. In the second step, the evolutionary selection step takes place. Here, the particles are *resampled* according to their weights. That is, a *new* set of particles is constructed by sampling from the set of particles $\theta _{t-1}$, proportional to the weights. This ensures that particles with high likelihood scores get to propagate to the next iteration, while particles with poor scores are taken out of the collection. The newly sampled values form the particles at iteration *t*.
3. Lastly, the particles are *mutated*, which proceeds by performing a series of *S* MCMC steps for each particle (see Section “Markov chain Monte Carlo approximations of the posterior”). The target density for this MCMC procedure is $p_t(\theta \mid D) \propto p_{t}(D\mid \theta )p(\theta )$. Note again the subscript *t* in the target density; it is possible (and useful) that these target densities change as the SMC algorithm proceeds; only at the last iteration is this target density equal to the posterior distribution we were ultimately looking for.

**Fig. 1:**
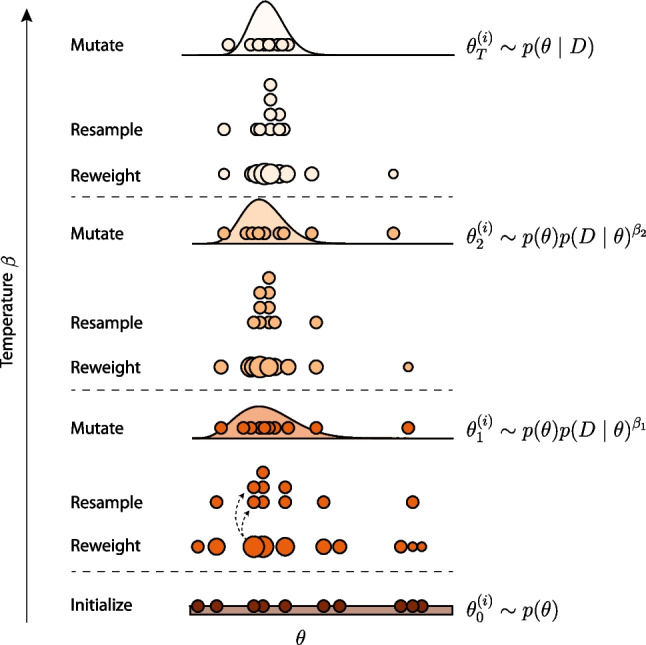
Visual representation of the adaptive-tempered Sequential Monte Carlo algorithm. Shown are $T=3$ iterations. The initial particles $\theta _0^{(i)}$ are drawn from the prior. Next, the weight of each particle is determined and indicated here using the relative size of each dot. Then the particles are resampled proportional their weights. Particles with large weights will result in multiple resampled particles. For example, the *dashed arrows* indicate a particle that is resampled twice. Lastly, particles are mutated by MCMC sampling using the tempered distributions as their target. This process is repeated until $\beta _T = 1.0$, at which point the distribution of particles follows the posterior distribution

The set of particles at the final iteration of the algorithm are used to approximate $p(\theta \mid D)$. A visual representation of the algorithm is shown in Fig. 1.

A key property of SMC is that the mutation procedures of the *M* particles are independent of one another, within one SMC iteration. At the same time, the computations that are performed between each iteration (the re-weighing and re-sampling) are computationally cheap. This means that the time-consuming particle mutation steps can be performed in parallel. This makes it possible, roughly speaking, that an SMC algorithm evaluates up to *M* times as many values for $\theta$ in the same amount of wall-clock time as an MCMC algorithm (assuming that within the mutation step we use the same MCMC algorithm).

As we will see later, the mutation step is crucial in ensuring the particles provide useful samples of the posterior distribution and meaningful contributions to marginal likelihood estimates. We investigate the impact of different common MCMC algorithms for the mutations in our applications in Section “Applications”.

### Improvements: Adaptive tempering

A naive implementation of SMC might run into a problem known as *particle collapse* (or *particle degeneracy*): If we resample according to the particle weights, we end up with a new set of particles in which the high-weight particles of the previous iteration are over-represented. After a few of those steps, all the particles become identical; rather than exploring the target density, we have only identified a single high-likelihood value. To prevent this, one can use a tempered variant of SMC (T-SMC) (Jasra et al., 2011; Speich et al., 2021).

The intuition for tempering is as follows. Rather than sampling from a target distribution $p(\theta \mid D) \propto p(D\mid \theta )p(\theta )$, we initially ignore the likelihood entirely. This means the random walks for each particle will simply explore the (wide) prior distribution. We then, in each SMC iteration, slowly increase the influence of the likelihood, steadily constraining the random walk behavior, until at the final iteration, the particles’ random walk does follow the posterior. The advantage is that the particles are less likely to collapse on one high-likelihood value. Simultaneously, tempering is helpful when using SMC to estimate the marginal likelihood of a model, which we cover in more detail in Section “Marginal likelihood estimation”.

We proceed with a more rigorous exposition of tempered SMC. In T-SMC, we associate a temperature parameter $\beta _t$ with each SMC iteration *t*, and we restrict $\beta _1=0< \beta _1 < \ldots \beta _T=1$. The temperature is used to dampen the influence of the likelihood on the target density at a specific iteration:

6 $$\begin{aligned} p_t(\theta \mid D) \propto p(\theta ) p(D \mid \theta )^{\beta _t} . \end{aligned}$$

The tempered density $p_t(\theta \mid D)$ is also referred to as a ‘bridge’ density (Herbst & Schorfheide, 2014). In the first iteration, where we have $\beta =0$, the bridge density simply is the prior distribution, while in the final iteration, the bridge density has become the posterior distribution. The particle weights after each SMC iteration are computed as

7 $$\begin{aligned} w_t^{(i)} = \frac{ p\left( D \mid \theta _{t-1}^{(i)}\right) ^{\beta _t}}{ p\left( D \mid \theta ^{(i)}_{t-1}\right) ^{\beta _{t-1}}} = p\left( D \mid \theta _t^{(i)}\right) ^{\Delta \beta } , \end{aligned}$$

where $\Delta \beta _t = \beta _t - \beta _{t-1}$ is the difference in temperature between consecutive iterations. As a result of this tempering, the variance between the weights decreases, which makes it less likely that one particle completely dominates all others.

The rate at which the temperature is updated from 0 to 1 needs to be set by the user, and could, for example, follow a linear or exponential scheme. An alternative to a predetermined tempering schedule is an adaptive approach, in which the increase in temperature between successive iterations is a function of the diversity of particle weights. This can be quantified in different ways, such as via the effective sample size (Agapiou et al., 2017):

8 $$\begin{aligned} M_{\text {eff}}(\textbf{w}_t, \Delta \beta ) = \frac{\left( \sum _{i=1}^M w_t^{(i)}\right) ^2}{\sum _{i=1}^M \left( w_t^{(i)}\right) ^2} = \frac{\left( \sum _{i=1}^M p\left( D \mid \theta _t^{(i)}\right) ^{\Delta \beta }\right) ^2}{\sum _{i=1}^M \left( p\left( D \mid \theta _t^{(i)}\right) ^{\Delta \beta }\right) ^2}. \end{aligned}$$

The increase in temperature corresponding to a desired effective sample size is then determined by solving for the roots of $f(\textbf{w}_t, \Delta \beta ) = M_{\text {eff}}(\textbf{w}_t, \Delta \beta ) - \alpha M = 0$, where $\alpha$ is the fraction of particles that we want to be independent, often heuristically set to $\alpha =0.5$ (Chopin & Papaspiliopoulos, 2020; Herbst & Schorfheide, 2014). Solving the equation is done via numerical methods. Not only do such adaptive approaches relieve the user of determining the right tempering schedule but they have also experimentally been shown to outperform pre-defined schedules (Zhou et al., 2016). Throughout our examples, we will use this adaptive-tempered SMC approach.

**Algorithm 2:**
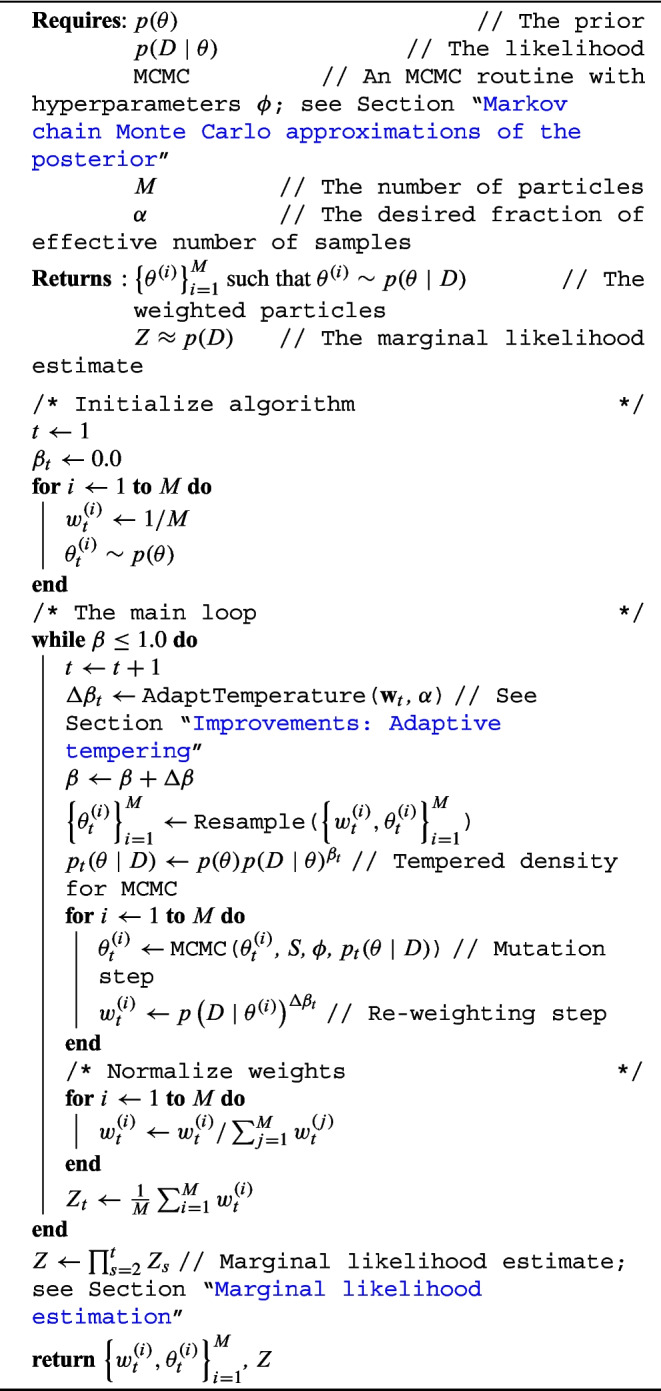
Pseudo-code for the adaptive-tempered Sequential Monte Carlo algorithm.

Pseudo-code of the SMC algorithm is provided in Algorithm 2. For clarity, we emphasize which elements should be provided by the user, assuming an SMC implementation is available. First, one should be able to evaluate the prior $p(\theta )$ and likelihood $p(D\mid \theta )$ for any value of $\theta$. Second, SMC assumes a subroutine is available for MCMC, that uses a starting position (the current particle) and returns a mutated value. This routine can have several hyperparameters, such as the number of MCMC steps to take, or the step size of a proposal distribution. Lastly, one needs to determine the number of particles *M*. Typically, more is better, but this depends on the available memory. We usually set $M=1000$. Finally, if we use the recommended adaptive-tempered SMC variant, then we need to set the fraction of effective samples $\alpha$. Typically, we set $\alpha =0.5$, representing that the effective sample size is half that of *M*.

### Marginal likelihood estimation

So far, we have focused on using SMC as an approximate inference algorithm, and therefore as an alternative to MCMC. However, the (adaptive) tempered SMC algorithm also provides an estimate of the marginal likelihood (Chopin & Papaspiliopoulos, 2020; Mlikota & Schorfheide, 2023; Zhou et al., 2016; Friel & Wyse, 2012), at negligible additional computation. The marginal likelihood quantifies how much evidence the observations provide for our model (c.q. hypothesis), and forms the basis of Bayesian hypothesis testing (Wagenmakers et al., 2018) (see Eq. (1)). Before we show how the SMC algorithm provides an estimate of this crucial quantity, we show the two most (seemingly) obvious ways to approximate the marginal likelihood.

The first simply replaces the integral in Eq. (1) with a Monte Carlo sum. This is known as the naive Monte Carlo approximation, and is defined as

9 $$\begin{aligned} p(D) =\int p(D\mid \theta ) p(\theta ) {\text {d}}\!{\theta } \approx \frac{1}{S} \sum _{i=1}^S p\left( D \mid \theta ^{(i)}\right) , \end{aligned}$$

with $\theta ^{(i)}\sim p(\theta )$. To obtain this estimate, we draw random values from the prior, and evaluate the likelihood for each of these. This estimator is asymptotically correct, and in the limit of $S\rightarrow \infty$ it will result in an unbiased estimate of the true marginal likelihood. However, it is often prohibitively inefficient. Since the prior support is usually much wider than the support of the posterior, it is unlikely that by sampling from the prior we encounter high-likelihood values. This means that most of the individual samples contribute very little to the marginal likelihood estimate, and that it is likely that we miss by chance those samples that would result in high likelihoods.

The second approach does the opposite, and uses samples from the posterior distribution instead (assuming these are available from MCMC, SMC, or another method entirely). This is known as the harmonic mean estimator, and is given by

10 $$\begin{aligned} p(D) \approx \left( \frac{1}{S} \sum _{i=1}^S \frac{1}{p\left( D \mid \theta ^{(i)}\right) }\right) ^{-1}, \end{aligned}$$

with $\theta ^{(i)}\sim p(\theta \mid D)$. One might think this estimator solves the problem of the naive Monte Carlo approach. After all, by sampling from the posterior instead, we definitely have high-likelihood samples in our collection. Unfortunately, this approach has been known to fail dramatically (Clyde et al., 2007), and has been dubbed the ‘worst Monte Carlo method ever’ by Neal (2008). The reason is that it often has infinite variance, even in toy models. For this estimator, the opposite happens as with the naive Monte Carlo method: This time, the high-likelihood values are over-represented (since the Monte Carlo samples come from the posterior), and the estimator does not properly integrate over the support of the prior.^3^

The SMC algorithm interpolates between these two approaches and approximates the marginal likelihood via

11 $$\begin{aligned}  p(D) & = \int p(D\mid \theta ) p(\theta ) {\text {d}}{\theta }\\ & \approx \prod _{t=2}^T \frac{1}{M}\sum _{i=1}^M p \left( D\mid \theta _t^{(i)} \right) ^{\Delta \beta _t}, \end{aligned}$$

with $\theta _t^{(i)} \sim p_t (\theta \mid D)$, and *T* the total number of SMC iterations (Chopin & Papaspiliopoulos, 2020; Zhou et al., 2016)^4^. This approach forms a middle ground between the two other approaches: In early stages of the adaptive SMC process (small *t*), the samples of $\theta _t^{(j)}$ are drawn from a tempered distribution that is dominated by the prior. This ensures the marginal likelihood properly integrates over the full prior support, just like the naive Monte Carlo approach. In later stages (large *t*), these samples are drawn from a distribution that closely resembles the posterior, thus contributing mostly the high-likelihood values. As a result, the estimator combines the best of both approaches.

Understanding the theoretical limits and convergence guarantees of adaptive-tempered SMC algorithms is challenging, since the estimates at different iterations depend on both the target density as well as the current (random) set of particles (Del Moral et al., 2012). For the standard SMC algorithm (without adaptive tempering), Chopin and Papaspiliopoulos (2020) show that it provides an unbiased estimate of the marginal likelihood, but this proof does not apply to the adaptive variants of SMC. Despite the lack of formal guarantees, Chopin and Papaspiliopoulos (2020) empirically find that the adaptive SMC algorithm similarly results in a consistent and well-behaved estimator. We return to this topic in our experiments.

**Fig. 2:**
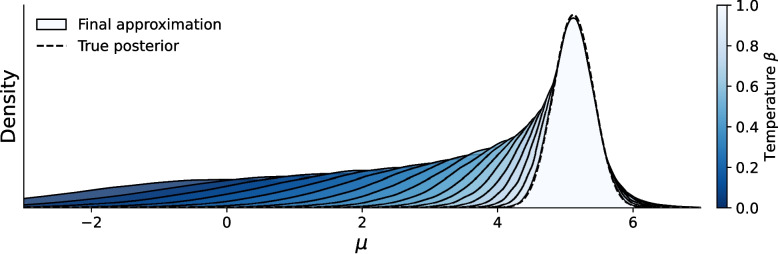
Approximate inference using Sequential Monte Carlo on a model with a Gaussian likelihood and a (conjugate) Gaussian prior on the mean. Superimposed on the approximated distribution is the exact solution (*dashed line*), which can be derived analytically from the conjugacy. The* shaded distributions *show the tempered distributions at different iterations of the SMC algorithm

### Toy example

The following toy example demonstrates the SMC algorithm in action. We assume a simple scenario consisting of a conjugate likelihood and prior pair, so that we can compare the SMC approximation with a ground truth. First, we simulate $N=100$ observations $\textbf{y}=(y_1, \ldots , y_N)^\top$, from a Gaussian distribution with parameters $\mu =5$ and $\sigma =3$. We then try to recover the true posterior $p(\mu \mid \textbf{y})$ from the observations, as well as the marginal likelihood $p(\textbf{y})$ using the following conjugate model:

12 $$\begin{aligned} \mu  &\sim \mathcal {N}\left( \mu _0, \sigma ^2_0\right) \\ y_i  &\sim \mathcal {N}\left( \mu , \sigma ^2\right) , \qquad i=1, \ldots , N , \end{aligned}$$

where we set $\mu _0=0.0, \sigma _0=2.0$. We assume the standard deviation $\sigma =3.0$ is known.

Because the Gaussian prior on $\mu$ is conjugate to the likelihood, the posterior distribution $p(\mu \mid \textbf{y})$ is once more a Gaussian distribution. However, we will pretend approximate inference is necessary, and run the SMC algorithm. This requires we provide first the likelihood and prior as given above. Furthermore, we use the Metropolis–Hastings MCMC algorithm as described in Section “Markov chain Monte Carlo approximations of the posterior” to mutate the particles, using a Gaussian proposal distribution with a standard deviation of 0.01 and $S=1000$ mutation steps per SMC iteration. We choose $M=100,000$ particles.

Figure 2 shows the resulting approximated posterior together with the true exact posterior distribution. Also indicated are the intermediate bridge densities that correspond to temperatures $\beta _t < 1.0$. The algorithm took 18 adaptive SMC cycles to increase the temperature from 0 to 1. As the figure shows, the algorithm gradually warms up and becomes increasingly more influenced by the likelihood. When the final temperature is reached (that is, $\beta _t=1.0$), the approximation agrees nearly perfectly with the true posterior. Similarly, the SMC algorithm closely approximates the marginal likelihood, estimating it at $-261.156$, compared to the true value of $-261.157$.

### Evaluating the posterior

When using Markov chain Monte Carlo methods, the target distribution $p(\theta \mid D)$ is approximated by a finite set of samples. However, if the collected samples are mostly determined by the random initial conditions of the algorithm, they do not reflect the posterior. To ensure sufficient samples have been collected and that they actually represent the posterior, one typically evaluates the collected samples according to heuristic criteria known as *convergence checks*. Once these are satisfactorily met, we say that for a sample with index *i*, $\theta ^{(i)}\sim p(\theta \mid D)$. Different measures and criteria exist to make this call.

A common convergence heuristic is based on the following intuition. First, we perform multiple runs of the MCMC algorithm, known as ‘chains’, that are all initialized uniquely. Once the distributions estimated by the different chains are sufficiently similar and are no longer determined by the initial condition, we assume they represent the desired distribution. This is quantified using the *potential scale reduction factor* (PSRF) $\hat{R}_\theta$, which measures the ratio of between-to-within chain variance for each variable in $\theta$ (Gelman & Rubin, 1992; Brooks & Gelman, 1998). A heuristic threshold is applied to this score, often set to 1.1, so that if $\hat{R}_\theta < 1.1$ for each variable $\theta$, we assume convergence has been attained.

While for MCMC methods convergence measures like the PSRF are well-established, determining similar criteria for SMC is an area of ongoing research (Dai et al., 2022; Beskos et al., 2016). Unfortunately, just completing the adaptive-tempering procedure (thus reaching $\beta =1$ at which point the obtained samples allegedly come from the posterior) does not guarantee convergence in practice. As a pragmatic solution for the convergence checks, we therefore use the following procedure in the examples below: we re-run multiple independent runs of the SMC algorithm with an increased number of mutations per SMC cycle, $S_t$, until the final collected samples have converged according to the PSRF. In contrast to MCMC, this requires restarting the algorithm, instead of appending subsequent samples to the previously collected samples, which is much less efficient.

Once convergence is established, it is common practice to determine, per variable, the *effective sample size* (ESS). Different from the effective sample size $M_{\text {eff}}$ that was used in Eq. (8) to quantify particle diversity, here the ESS gives a measure of how independent the samples of our approximation are. For example, if we had collected 1000 samples with an MCMC algorithm, but found an ESS of 200, then we would have essentially obtained the information equivalent of only 200 independent samples. This increases the uncertainty in subsequent Monte Carlo estimates. In the context of SMC, autocorrelated samples should in theory pose less of an issue than for MCMC, since rather than a single Markov chain (which is autocorrelated by definition), we have different particles that individually mutate. Still, particles may spawn from the same parent particle at the previous SMC iteration, so autocorrelation can be present here as well. In the applications below, we quantify this by computing the ESS for different inference algorithms. For details on the computation and implementation of the ESS, we refer to Gelman et al., (2013).

## Applications

After this introductory exposition of the Sequential Monte Carlo algorithm, we proceed here to give three worked examples of Bayesian models one might use in practice, and showcase the practical usefulness of SMC. Each of the examples highlights a different feature of SMC: its efficiency in approximating a posterior distribution, the ability to deal with multimodal distributions, and the quality of its marginal likelihood estimate. Each of these forms the focus of one of the examples below.

### Inference: Variable selection in depression

The first example shows how the posterior estimates obtained with SMC agree with popular MCMC algorithms.

We consider the problem of variable selection. This is a commonly encountered challenge in psychology, and is used, for instance, to improve the quality of screening instruments for psychiatric disorders (Lu & Petkova, 2014; Akyol, 2020), to identify predictors of psychopathology (Meehan et al., 2020), and to learn which factors influence a person’s mental well-being after experiencing stressful events (Liu et al., 2021). Here, we use variable selection to determine which factors are associated with depression.

The core of the model is linear regression, with regression coefficients corresponding to each of these factors. Let *N* be the number of observations, for which we observe the values for *p* predictors $\textbf{x}_i=(x_{i1}, \ldots , x_{ip})^\top$, as well as the corresponding response variable $y_i$. We aggregate the predictors in a matrix $\textbf{X}=(\textbf{x}_1, \ldots , \textbf{x}_N)^\top$, and similarly we collect all responses into the vector $\textbf{y}=(y_1, \ldots , y_N)^\top$. The regression model is given by

13 $$\begin{aligned}  \log \sigma&\sim \mathcal {N}(0, 1) , &  \\ \beta _j&\sim p(\beta _j), &  j=1, \ldots , p \\ y_i \mid \varvec{\mathbf {\beta }}, \textbf{x}_i, \sigma&\sim \mathcal {N}(\textbf{x}_i^\top \varvec{\mathbf {\beta }}, \sigma ^2), &  i=1, \ldots , N.  \end{aligned}$$

Several candidate variable selection distributions exist for the prior on the coefficients $p(\beta _j)$ (O’Hara & Sillanpää, 2009). Common choices include a Gaussian or a Laplace distribution, the regularized horseshoe distribution (Piironen & Vehtari, 2017), or spike-and-slab distributions (Malsiner-Walli & Wagner, 2017). All of these distributions encourage the values for the regression coefficients to be small or even exactly zero, which implies that the corresponding predictor is not relevant for the prediction of depression. The choice for a particular prior can be motivated by several reasons, such as the interpretation of additional model parameters, or pragmatic considerations. For example, if one uses the popular NUTS algorithm for inference (Hoffman & Gelman, 2014), then one of the requirements of this approach is that the model does not contain discrete latent variables, since the algorithm depends on the computation of gradients.

Here, we use the logit-normal continuous analogue of the spike-and-slab (Thomson et al., 2019) (LN-CASS) prior on the coefficients. It is, as the name implies, a continuous distribution with strong conceptual similarities to the discrete spike-and-slab distribution (George & McCulloch, 1993). The advantage of this particular prior is that it consists only of continuous variables, as opposed to spike-and-slab distributions, which makes it amenable to gradient-based MCMC approaches such as NUTS, while maintaining the conceptual interpretation of the spike-and-slab. The full mathematical definition of the prior is provided in Appendix A.1.

#### Data

Our observations consist of a sample ($N=715$) from the general population taken from the Nathan Kline Institute Rockland Sample (Nooner et al., 2012), a publicly available dataset aimed at improving scientific research into psychiatry. A large number of self-reported measures are available for these participants. We follow the setup described by Bainter et al. (2023) and include the following potentially relevant predictors (the abbreviated name for the corresponding predictors is shown in parentheses): four subscales of the Adult Temperament Questionnaire (ATQ) (Evans & Rothbart, 2007), four subscales of the Interpersonal Reactivity Index (IPRI) (David, 1983), five subscales of the Domain Specific Risk Taking Scale (DOSP) (Blais & Weber, 2006), five subscales of the Urgency, Premeditation, Perseverance, Sensation-Seeking and Positive Urgency impulsive behavior scale (UPPS) (Whiteside & Lynam, 2001), the total sleep quality score from the Pittsburgh Sleep Quality Index (PSQI) (Buysse et al., 1989), the Fagerström Test for Nicotine Dependence (Nicotine) (Heatherton et al., 1991), and the total time that a person is physically active using the International Physical Activity Questionnaire (IPAQ) (Craig et al., 2003). In total, $p=24$ predictors are collected. Finally, the targets for prediction $\textbf{y}$ were the depression symptom severity as measured by the Beck Depression Inventory II (BDI-II) (Beck et al., 1996). A list of the variable names is provided in Appendix A.3.

#### Comparing different approximate inference methods

We perform the approximate inference of the posterior distribution using six distinct approaches. The first are three MCMC-based methods: A Metropolis–Hastings MCMC algorithm (MH; see Section “Markov chain Monte Carlo approximations of the posterior”) with Gaussian proposals, a block-Gibbs sampler, in which individual variables are updated in turn, using a separate MH step for each, and an adaptively tuned No-U-Turn-Sampling Hamiltonian Monte Carlo (NUTS) algorithm (Hoffman & Gelman, 2014), which can be considered the state-of-the-art. The second three methods consist of the adaptive-tempered Sequential Monte Carlo (SMC) algorithm as described in the previous section, combined with each of the three MCMC approaches for the mutation step. The details of the inference settings are provided in Appendix A.2. In each case, we ran four independent chains with as many MCMC steps as required until convergence was reached, quantified using the potential scale reduction factor heuristic (Gelman & Rubin, 1992) (see Section “Evaluating the posterior”).

**Fig. 3:**
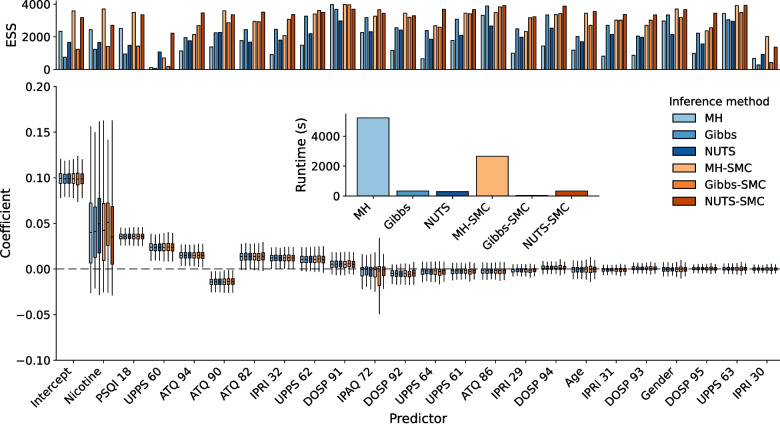
Estimated regression coefficients using Bayesian linear regression together with the LN-CASS prior. Predictors are sorted by their mean absolute value. The most important predictors are nicotine and sleep quality. All six methods result in the same posterior distributions, although the number of effective samples that they obtain differ (*see top row*). Using SMC improves the number of effective samples, while at the same time reducing wall clock running time. The Gibbs-in-SMC algorithm is by far the fastest, and results in the highest number of effective samples per second

Figure 3 shows the estimated regression coefficients using the LN-CASS prior. Importantly, the approaches provide identical estimates. All six identify the same ordering of importance of the individual factors, with nicotine dependence and sleep quality being among the most important predictors, although the former predictor is associated with a large amount of uncertainty. Furthermore, the inset bar chart shows the (average) relative wall-clock time that was required for the algorithms to converge. Both Gibbs and NUTS are much more time-efficient than the Metropolis–Hastings MCMC algorithm, but MH and Gibbs benefit most from being embedded in the larger SMC routine; their computation times drop drastically (from 5226 to 2654 seconds for MH, 329 to 45 seconds for Gibbs), while their effective numbers of samples increase. This benefit does not apply to NUTS, where the additional computation time does not outweigh the benefits of SMC; both NUTS and NUTS-in-SMC take about the same time (299 seconds for NUTS HMC compared to 336 when embedded within SMC), although the number of effective sample size also goes up substantially when using NUTS within SMC. The SMC routine takes 47.25, 51.75, and 47 adaptive cycles, for MH, Gibbs, and NUTS mutations, respectively, showing that type of mutation algorithm has only a limited impact on the number of SMC cycles. The conclusion of this first demonstration is that SMC is an effective approximate inference tool, with the potential to reduce running time and increase effective sample sizes substantially.

### Multimodality: A growth mixture model for grade point averages

In this second example, we consider mixture models and the challenges they provide for approximate inference algorithms. With the advent of increasingly larger data sets, it is becoming clear that modeling heterogeneous populations with a single parametric distribution does not do justice to the complex structure in these data (Moreau & Corballis, 2019; Gao et al., 2023; Feczko et al., 2019). Instead, often a population consists of the combination of differently distributed sub-populations, which can be represented using *mixture models* (Harring & Hodis, 2016). For example, Bak et al. (2017) use a Gaussian mixture model to identify two distinct groups of patients suffering from schizophrenia. Similarly, Abu-Akel et al. (2019) use a Weibull mixture model to accurately represent the heterogeneity in a population of people with autism spectrum disorder, and Mora et al. (2008) use growth mixture models to distinguish different patterns in the timing and severity of depression symptoms of women with perinatal depression.

Here, we consider a Bayesian growth mixture model (GrMM; Ram and Grimm, 2009) to model the progress of grade point averages (GPA) for college students as they progress through the semesters of their studies. The intuition behind such a model is that we expect groups of students to progress in different ways; such as a group consisting of students that quickly progress in terms of their GPA, versus a group of students that perform more or less the same across the different semesters.

#### Data

The data are available from the website of the statistical analysis software JASP (JASP Team, 2024) and were originally collected by Hox (2010). The data consist of the GPAs measured at $T=6$ consecutive semesters, for $N=200$ college students. The GPA scores range from 1.7 to 4.0.

#### Model

In a growth mixture model, a single observation *i* is a time series $\textbf{y}_i=(y_{i1}, \ldots , y_{iT})^\top$ of length *T*, with $i=1,\ldots , N$. Stacking these columns together results in the matrix $\textbf{Y} \in \mathbb {R}^{N \times T}$. Corresponding to these observations, we have the locations (time points) as the vector $\textbf{x}_i =(x_{i1}, \ldots , x_{iT})^\top$. Throughout this example, we assume that all observations are performed at the same input locations, so we are only concerned with a single vector of locations $\textbf{x}$.

We assume the different students are not independent, but are clustered instead; each student is assigned to a latent mixture component using the variable $z_i \in \{1, \ldots , K\}$, with *K* the number of these components. In practice, this discrete variable can be marginalized out to result in a model that is more amenable to inference. The likelihood of such a model is

14 $$\begin{aligned} p\left( \textbf{Y} \mid \textbf{x}, \varvec{\mathbf {\theta }}\right) = \prod _{i=1}^N p\left( \textbf{y}_i \mid \textbf{x}, \varvec{\mathbf {\theta }}\right) , \end{aligned}$$

where $\varvec{\mathbf {\theta }}$ represents the collection of latent parameters of the model, and with the probability of a single point $\textbf{y}_{i}$ given by

15 $$\begin{aligned} p\left( \textbf{y}_i \mid \textbf{x}, \varvec{\mathbf {\theta }}\right) = \sum _{k=1}^K w_k \prod _{t=1}^T \mathcal {N}\left( \sum _{d=0}^D \beta _{dk} x_t^d, \sigma ^2_k \right) . \end{aligned}$$

In the last expression, we see that each of the *K* components brings their own contribution to the likelihood. Within each component, each observation at time point *t* is assumed to follow a Gaussian distribution. The summation over *D* indicates the degree of the polynomial that is used to model the component growth curve. Here, we simply set $D=1$, representing each component’s growth with a linear curve based on an intercept $\beta _{0k}$ and a slope $\beta _{1k}$. The model is completed by specifying the priors on the latent parameters $\varvec{\mathbf {\theta }}$, which are the component weights $\textbf{w}$, the growth curve regression coefficients $\textbf{B}=\{\beta _{dk}\}$, which are specific to each mixture component, and the different observation noise terms $\varvec{\mathbf {\sigma }}=(\sigma _1, \ldots , \sigma _K)^\top$. The choices for these priors are provided in Appendix B.1.

**Table 1:** Evaluation of the six different inference approaches (indicated are mean and standard error over four chains, when applicable)

Algorithm	PELL	KL	ESS	Time (s)
MH	$-158.45 \pm 4.00$	$12.22 \pm 1.55$	4.94	456.53
Gibbs	$-157.98 \pm 2.92$	$11.04 \pm 2.18$	4.16	550.91
NUTS	$-156.45 \pm 0.27$	$14.73 \pm 0.16$	2.15	242.05
MH-in-SMC	$-157.20 \pm 0.29$	$2.02 \pm 1.18$	19.66	173.04
Gibbs-in-SMC	$-157.30 \pm 0.61$	$0.64 \pm 0.13$	17.77	40.22
NUTS-in-SMC	$-156.84 \pm 0.40$	$0.32 \pm 0.16$	69.75	303.27

The top row shows the posterior expectation of the log-likelihood (PELL) for the growth mixture model with $K=4$ components (larger is better). The results indicate that in terms of predictive performance, the approaches perform nearly identically. However, the multimodality is captured much better by the SMC-based approaches, as shown by the Kullback–Leibler divergence (KL). Since the different chains are more consistent for SMC, the effective sample size (ESS) is higher as well. At the same time, SMC converges faster than MCMC in wall-clock time, with Gibbs-in-SMC being particularly quick

Learning the growth mixture model consists of estimating the posterior $p(\textbf{w}, \textbf{B}, \varvec{\mathbf {\sigma }} \mid \textbf{x}, \textbf{Y})$. However, approximating this distribution is less straightforward than it may seem. The distribution is multi-modal, which means that it contains different areas in the posterior distribution with the same probability. In general, such multimodality can occur due to different reasons, such as model misspecification leading to problems with identifiability, or simply different valid parameter configurations that are all supported by the observations, but that represent qualitatively different posterior beliefs. In mixture models, this is the case as well. Any permutation of the labels of the different mixture components would result in the exact same probability density, which leads to multiple configurations of parameter values with the same probability. For instance, switching all parameters associated with component 2 with all of those associated with component 3 leaves the density unchanged, and so forth. This issue is known as *label switching*, and can hamper effective mixing of MCMC algorithms: the sampler will remain stuck in one configuration of the labels, and will not explore the others. In specific cases, the multimodality could simply be removed by imposing an ordering constraint on some of the parameters. For instance, if we assume $\beta _{j1}< \ldots < \beta _{jK}$, the labels of the components could no longer be switched without changing the posterior density. However, in higher dimensional mixture models, it is not obvious how the elements can be similarly constrained. For the purpose of the illustration here, we deliberately leave the multimodality present so that we can evaluate the comparative performance of the inference algorithms in this difficult scenario.

#### Experiment

Once more, we apply three MCMC-based algorithms and three MCMC-within-SMC algorithms to estimate the posterior distribution of this model. For this example, we pick $K=4$, although one might use the marginal likelihood to determine the optimal *K*.

Determining the required number of MCMC samples and evaluating the results proceeds differently compared to the previous example. A regular convergence check is difficult here, as multimodality causes independent chains to become stuck in different modes of the posterior. Even though the algorithms would be sampling high-probability parameter values, the chains would seem very distinct, so the potential scale reduction factor heuristic would lead us to conclude that the algorithms have not converged. In fact, convergence issues can often be attributed to multimodality and unidentifiability issues. As an alternative, we track the posterior expectation of the likelihood (PELL), that is

16 $$\begin{aligned} \mathbb {E}_{p(\theta \mid \textbf{x}, \textbf{Y})}\left[ \log p\left( \textbf{Y}\mid \textbf{x}, \theta \right) \right] \approx \frac{1}{S} \sum _{i=1}^S \log p\left( \textbf{Y}\mid \textbf{x}, \theta ^{(i)}\right) , \end{aligned}$$

$\theta ^{(i)}\sim p(\theta \mid \textbf{x}, \textbf{Y})$ and see for how many MCMC samples or SMC mutation steps this value is within 2% of the result of a long adaptively tuned NUTS approximation.

Once we are satisfied with convergence, we look at the recovery of the multimodal posterior by comparing the distribution of $p(\beta _{1k} \mid \textbf{x}, \textbf{Y})$, that is, the slope of the linear growth curve, across the different components. If the multimodality is captured correctly, the distributions of $p(\beta _{11} \mid \textbf{x}, \textbf{Y})$, up to $p(\beta _{1K} \mid \textbf{x}, \textbf{Y})$ should be indistinguishable, as the different permutations of label switching should all be represented. If instead the algorithm is stuck in a single mode, then these distributions can differ wildly. We quantify the similarities between these distributions using the Kullback–Leibler divergence (see Appendix B.3 for details on its computation). Finally, we collect the running time of each algorithm.

Table 1 shows the results of the analysis. The algorithms reach similar predictive performance, showing that they are exploring high-probability areas of the posterior. However, from both the Kullback–Leibler divergence and the effective sample sizes, it is apparent that MCMC-based methods quickly find a local mode and have trouble leaving it; the distributions of the coefficients of the different mixture components remain clearly separated. For SMC this is not the case, as shown by the much lower KL divergence. This means the different components have very similar posterior distributions, indicating that the label switching is accounted for. At the same time, SMC generally converges more quickly, especially in the case of Gibbs where a more than tenfold speed increase is observed. Similar to the previous example, we see that the additional computation required for NUTS offsets the benefits of SMC, as NUTS-within-SMC takes longer to converge than NUTS on its own. However, the effective number of samples shows that NUTS-within-SMC provides the best posterior representation. The number of required SMC cycles is stable, with on average 38.5, 38.75, and 39.25 for MH, Gibbs, and NUTS as mutation algorithm, respectively.

**Fig. 4:**
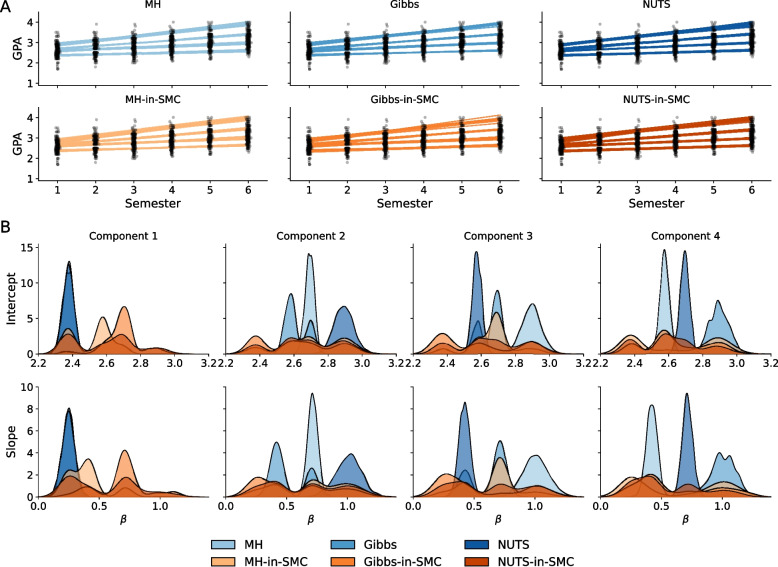
Posterior distribution of the growth mixture model with $K=4$ components and $D=1$, that is, linear regression per component. **A** The fit of the posterior distribution to the observations, for each of the six approximate inference methods: Metropolis–Hastings MCMC, Gibbs MCMC, NUTS HMC, and SMC with each of these MCMC algorithms in the mutation step. **B** Posterior distributions of the intercepts and slopes for each of the *K* mixture components. If the multimodality was captured perfectly, the distributions in each panel of a row would be similar and showing evidence of multiple modes. Although no method performs perfectly, SMC covers several of the modes, while the three MCMC-based alternatives are stuck in individual modes

In Fig. 4A, the posterior fit of the growth mixture model is superimposed on the observations. This figure confirms that all inference approaches find a solution that fits the data well, and that they all agree on what this solution is. However, Fig. 4B shows the posterior distributions of the regression coefficients for each of the mixture components, that is, $p(\beta _{0k} \mid \textbf{x}, \textbf{Y})$ and $p(\beta _{1k} \mid \textbf{x}, \textbf{Y})$, for $k=1, \ldots , K$, and these are quite distinct. This demonstrates how SMC is able to represent several of the different modes in this posterior distribution, while the MCMC-based algorithms are stuck in isolated modes (that is, in one of the peaks of high probability density near 2.4, 2.6, 2.7, and 2.9 for the intercept $\beta _0$, and around 0.25, 0.4, 0.7, and 1.0 for the slope $\beta _1$). Despite this improved representation of the multimodal posterior, it should be noted that such distributions and their approximations should still be treated with caution. In particular we see that the different modes are not completely uniformly distributed over the particles; some modes seem to be preferred over others. This might erroneously suggest that one mode is more likely than another, while in fact this is probably due to a random fluctuation in which particle ends up in which node. Increasing the number of particles will reduce this problem, but at the cost of additional computational resources.

Although in cases like this the multimodality can be avoided by imposing ordering constraints on some parameters, this becomes much harder when the dimensionality of the data increases or higher-order regression functions are used. In other cases, we might not even be aware that the multimodality exists in our posterior, and we might have trouble reaching convergence. In such cases, SMC provides clear benefit by actually showing the multimodality in the posterior. Importantly, due to the parallel computation across particles, this benefit is obtained while converging faster than the other methods as well.

### The marginal likelihood: The expectancy-valence model

The previous examples highlighted SMC as an efficient and effective algorithm for approximate inference, even in the challenging case of multimodality. In this section, we demonstrate how SMC can be applied in the context of cognitive modeling, and we emphasize how SMC can be used to obtain an estimate of the log marginal likelihood together with the samples of the posterior distribution.

We consider the Iowa Gambling Task (IGT; Bechara et al., 1994) and the expectancy-valence model (Busemeyer & Stout, 2002) that is often used in conjunction with it, as this combination of task and model has been used previously to study the estimation of marginal likelihoods (Steingroever et al., 2016; Gronau et al., 2017) and therefore allows for a comparison with competitive methods. In the IGT participants are presented with four decks of cards, and the different cards in these decks are associated with either a reward or a penalty. The participants are instructed to sequentially pick cards from these decks that will maximize their total reward. The participants are not aware of the distributions of cards in the decks, nor of the fact that two decks contain high-reward cards, but in the long run will result in a lower profit than the decks containing moderate-reward cards. It is up to the participants to explore the reward distributions of the decks, and exploit these once they have an idea which decks lead to the best eventual result. For an overview of applications of the IGT, we refer to Aram et al. (2019).

A popular computational model for behavior of the IGT is the expectancy-valence (EV) model (Busemeyer & Stout, 2002). The model uses three types of observations from the IGT: the participant choice of a card deck at trial *t*, *k*, $k\in \{1, 2, 3, 4\}$, and the corresponding reward *W*(*t*) or penalty *L*(*t*). It then makes the following assumptions about the cognitive process of the participant: first, the participant has an internal representation of the utility $u_k(t)$ of deck *k*, which is updated based on the choice of deck, according to

17 $$\begin{aligned} u_k(t) = (1-w) W(t) + wL(t) . \end{aligned}$$

The first model parameter *w* determines the extend to which a participant is either reward-seeking (small values of *w*), or loss-avoiding (large values of *w*), and can be interpreted as the degree to which the participant is loss-oriented (Ahn et al., 2008.

Next, the EV model assumes that, based on the experienced utility $u_{kt}$, the *expected* utility $\mathbb {E}[u_{kt}]$ is updated using the Rescorla-Wagner rule (Rescorla & Wagner, 1972):

18 $$\begin{aligned} \mathbb {E}[u_k(t)] = \mathbb {E}[u_k(t-1)] + a \left( u_k(t) - \mathbb {E}[u_k(t-1)] \right) , \end{aligned}$$

with $a \in [0, 1]$. This equation implies that if the experienced utility is larger than what was expected ($u_k(t) > \mathbb {E}[u_k(t-1)]$), then the expectation is adjusted upward, and vice versa if the experienced utility is lower than expected. The parameter *a* is the second model parameter, and it determines the degree to which beliefs are updated based on the experienced utility. Here, a small value for *a* indicates that beliefs are strongly adhered to, and only weakly updated based on observed deviations. Alternatively, for values of *a* close to 1, the expected utility is updated radically depending on what was actually observed.

Subsequently, the model assumes that the updated set of expected utility determines which deck of cards the participant will select on their next trial. That is, the probability of deck *k* at the next trial is given by

19 $$\begin{aligned} \pi (t+1)_k = \sigma \left( \theta (t) \mathbb {E}\left[ u_k(t)\right] \right) _k , \end{aligned}$$

in which $\sigma (\cdot )$ is the softmax function defined as

20 $$\begin{aligned} \sigma (\textbf{w})_k = \text {softmax}(\textbf{w})_k = \frac{\exp (w_k)}{\sum _{k'=1}^K \exp (w_{k'})} . \end{aligned}$$

The sensitivity parameter $\theta (t)$ determines the degree to which the expected utility determines the probabilities of selecting each deck. For values close to zero, the term $\mathbb {E}[u_k(t)]$ has little effect, and choices are made at random, while larger values of $\theta (t)$ indicate that the participant bases their choices entirely on the expected utility. The sensitivity parameter $\theta (t)$ is not itself a free parameter of the model, but instead is derived from the third and final model parameter, known as the response consistency $c \in \mathbb {R}$, via the expression

21 $$\begin{aligned} \theta (t) = (0.1t)^c , \end{aligned}$$

which formalizes the assumption that the adherence to the acquired beliefs changes with time. If *c* is larger than 1, the successive choices become more and more determined by the expected utilities while negative values of *c* indicate the participant behaves in an increasingly random way, ignoring the expected utilities. Note that the domain constraint on *c* varies between studies, as Busemeyer and Stout (2002) assume $c\in [-5, 5]$, while Gronau et al. (2017) suggest that $c\in [-2, 2]$ provides more numerically stable parameter estimates. We follow the latter choice.

**Fig. 5:**
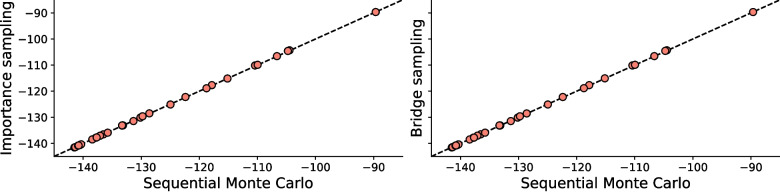
Log marginal likelihood estimates for the expectancy-valence model for $N=30$ participants using the data obtained by Busemeyer and Stout (2002). Shown are the estimates obtained with SMC, as well as with importance sampling (Steingroever et al., 2016) and bridge sampling (Gronau et al., 2017). As the scatter plots show, the estimates are virtually identical

Together, the reward-seeking versus reward-averse parameter *w*, the belief update parameter *a*, and the response consistency parameter *c* form the latent variables of the model, and their distributions are to be conditioned on the participants’ choices of decks, as well as the rewards and penalties received by the participants.

#### Data

We train the expectancy-valence model on observations collected by Stout et al. (2001); Busemeyer and Stout (2002). These data were studied previously in the context of marginal likelihood estimation by Steingroever et al. (2016) using importance sampling, and by Gronau et al. (2017) using bridge sampling, and therefore serve as an ideal comparison. The data consist of $T=100$ card deck selections and their corresponding rewards and/or penalties for $N=30$ healthy participants.

#### Training and evaluation of the EV model using Sequential Monte Carlo

We first estimate the log marginal likelihoods of the EV model for each of the 30 participants independently. The priors were chosen to be uninformative (see Appendix C.1 for more details), and we set the initial expected utility $\mathbb {E}[u_k(0)]=0$, for $k \in \{1,2,3,4\}$. We used 1000 particles and each of the three different MCMC algorithms for the mutations steps; Metropolis–Hastings, Gibbs, and NUTS. Details on the inference settings are provided in Appendix C.2. The desired fraction of effective samples in the adaptive tempering scheme was set to $\alpha =0.5$. The results for the log marginal likelihoods are compared with those obtained by importance sampling (Steingroever et al., 2016) and bridge sampling (Gronau et al., 2017), and this comparison is shown in Fig. 5. All three mutation kernels resulted in the same log marginal likelihood estimates, and therefore only the result for MH-in-SMC is shown. As the figure indicates, the log marginal likelihood estimates obtained by SMC are virtually identical to those from importance sampling and bridge sampling.

**Fig. 6:**
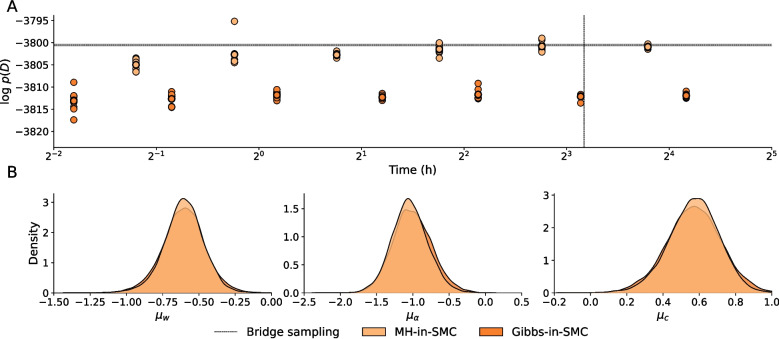
SMC estimation results for the hierarchical expectancy-valence model (Wetzels et al., 2010) **A**. The marginal likelihood estimates for different numbers of mutation steps, for MH-in-SMC and Gibbs-in-SMC. The mean estimate across the ten chains is shown as a *dot with a thick border*. The log marginal likelihoods obtained by bridge sampling (Gronau et al., 2017) are shown by the *gray interval* and *dashed line*. **B** The marginal posterior distributions for the three hierarchical mean parameters, $\mu _w$, $\mu _\alpha$, and $\mu _c$. The plotted distributions are the result of a kernel density estimate on the final 1000 particles

#### A hierarchical extension of the EV model

The previous section showed that Sequential Monte Carlo obtains reliable estimates of marginal likelihoods for cognitive models. However, the individual models contained only three latent parameters. We now turn to case of a hierarchical formulation of the expectancy-valence model (Wetzels et al., 2010; Gronau et al., 2017), which contains many more parameters, and is therefore much more challenging.

In the previous setup, the individual participants were considered to be conditionally independent, which means that the SMC algorithm was ran *N* times. In a hierarchical formulation, we instead assume a shared structure between the parameters of each individual. That is, we define a group-level mean and standard deviation for *w*, *a*, and *c*, and use these to inform the priors on the participant-specific parameters $w_i$, $a_i$, and $c_i$, for $i=1, \ldots , N$ (a detailed description of this hierarchical prior is provided in Appendix C.1). This way, the prior distribution of each participant-level parameter is informed by the $N-1$ other participants, which tends to result in much more robust parameter estimates (Kruschke, 2014). Due to this coupling of parameters, however, we now need to estimate all $6+3N$ parameters in one single distribution, which results in a 96-dimensional posterior. Here, we demonstrate that SMC can also be used in such high-dimensional model settings, and we explore what the effect of the number of mutation steps is on both the posterior and log marginal likelihood estimates (Dai et al., 2022).

We estimate the posterior and log marginal likelihood of the hierarchical EV model using SMC, using 1000 particles, using both MH-in-SMC and Gibbs-in-SMC. The NUTS-in-SMC approach turned out to be too slow to be practical, and therefore is omitted from this example. Since the number of mutation steps has substantial impact on the estimated marginal likelihood, we doubled the number of mutation steps until the marginal likelihood was similar to that obtained via bridge sampling, which we use as a gold standard here (more details on this step are provided in Appendix C.3). This procedure is repeated ten times, so that we obtain both a measure of the reliability of the log marginal likelihood estimates, as well as an indication of convergence of the approximation of the posterior.

The results for the marginal likelihood estimates, as well as the approximate posterior at the final number of mutation steps, are shown in Fig. 6. Regardless of the number of mutations, the MH-in-SMC takes approximately 41 adaptive SMC cycles, while Gibbs-in-SMC takes about 45 cycles. The approximation of the posterior requires only a limited number of mutation steps, as the potential scale reduction factor $\hat{R}$ drops below 1.1 for 500 MCMC steps for MH-in-SMC, and within 100 steps for Gibbs-in-SMC. The marginal likelihood estimate takes considerably longer to converge. Importantly, however, we see that or MH-in-SMC, the mean of the marginal likelihood estimates agrees with bridge sampling after about 32,000 mutations (7 h), and its variance reduces further when increasing the number of mutation steps to 64,000 (14 h). For comparison, the computation time for inference and bridge sampling is shown in the figure as well (9 h).

Perhaps surprisingly, Gibbs-in-SMC does not appear to converge to the correct marginal likelihood, at least not for these numbers of mutation steps. The reason that Gibbs-in-SMC works so well for inference (as seen in the previous examples) and for the marginal likelihood estimates in the smaller EV model, but not here, is presumably that the step sizes for the individual parameter updates work well at higher temperatures (that is, $\beta$ approaching 1), and thus leading to good approximations of the posterior, while accepting much fewer samples when the temperature is low (when $\beta$ is small and the bridging distribution is closer to the prior), and consequently integrating more poorly over the prior. The same observation was made by Buchholz et al. (2021), who conclude that for high-dimensional models, ideally the mutation step parameters should be adapted during the tempering process, so that acceptance rates are appropriate for all temperatures of the SMC algorithm.

The results for the independent and hierarchical variants of the expectancy-valence model demonstrate that SMC can approximate marginal likelihoods, even when the model is high-dimensional and contains many correlated parameters, as in the hierarchical case. The marginal likelihood estimates up to par with bridge sampling, albeit with the caveat that the quality is dependent on the quality of the mutation steps. Future work into adaptive proposal distributions to improve mixing at all temperatures will most likely improve the quality of these estimates further.

## Discussion

In this tutorial, we have discussed how Sequential Monte Carlo may be used as an alternative approach for approximate inference, as well as (possibly simultaneously) a method to estimate the marginal likelihood of a model. At the core of SMC lies the parallel execution of a large number of MCMC samplers, that are reweighed and resampled at every iteration of the algorithm. This parallelism brings a number of important benefits over existing approximate inference approaches such as MCMC. First, it enables the algorithm to more effectively explore the target distribution. In the first example, we saw that with the right MCMC mutations, SMC can be both faster as well as more providing more effective samples than conventional MCMC algorithms. Furthermore, the example on growth mixture models demonstrated that compared to standard MCMC samplers, SMC can obtain comparable model fit, while better representing the multimodality better and being faster in wall-clock running time. Of course, the latter comes with the caveat that in order to be efficient, SMC requires hardware that supports parallel computation, such as GPUs. While this is on one hand a constraint, as practitioners using SMC will need the required hardware, at the same time it provides a way to have the Bayesian framework leverage the nowadays ubiquitous parallel compute (Lee et al., 2010). Setting up SMC for inference takes hardly any additional effort over setting up MCMC, especially when the tempering schedule is determined adaptively. Two remaining choices are the number of particles and the desired effective sample size of the particle weights. The first choice directly determines the ‘resolution’ of the approximate posterior distribution. In our examples and experiments, we found that $1000--2000$ particles typically sufficed. If the posterior shows signs of multimodality, it is recommended to increase the number of particles further, so that each mode can have a reasonably smooth representation. For the second choice, we found that the default of 0.5 (that is, half of the total number of particles) worked well in all our examples.

Next to being a competitive approach for approximate inference, SMC, and in particular the tempered variant we have discussed in this tutorial, also provides a useful and effective way to estimate the marginal likelihood of a model. Importantly, this estimator requires very little effort from the end user. It suffices to provide the likelihood and the prior, and – depending on the MCMC step within each SMC cycle – a proposal distribution like one would provide in MCMC. We saw that the approach is competitive, even in high-dimensional cases such as the hierarchical expectancy-valence model (Busemeyer & Stout, 2002). The effectiveness of this estimator comes from the tempering procedure, which interpolates the target density between the prior (at low temperature) and the posterior (at high temperature). Concretely, this means that the Monte Carlo estimate of the marginal likelihood that SMC provides is based both on the entire support of the prior (in the early stages of the algorithm), as well as on parameter values with high likelihood (in the later stages of the algorithm). However, to obtain reliable estimates, a large number of mutation steps (that is, MCMC steps for each particle within a single SMC iteration) is needed, and this can impose substantial computational demands, as the algorithm duration scales linearly with this number. In the hierarchical expectancy-valence model, we saw that as many as 64, 000 steps were needed to obtain estimates similar to those obtained by bridge sampling. Furthermore, the better the MCMC algorithm in the mutation step, the more important it becomes to correctly set the step sizes of the proposal distributions. Here, a balance must be struck so that the mutation step explores reasonably well in both the prior and the posterior. In high-dimensional models, this can be challenging, and adaptation of the mutation kernels will be necessary, as discussed by Buchholz et al. (2021). Nevertheless, we believe that its ease-of-use and effectiveness make SMC a useful technique in the toolbox of researchers that need the marginal likelihood to perform model selection and model averaging (Clyde et al., 2011; Hinne et al., 2020).

There are, of course, also a number of downsides to SMC. The most obvious one is the aforementioned reliance on parallel computation hardware; if all computations were to be performed sequentially, the algorithm would be prohibitively slow. Furthermore, we saw that the quality of the marginal likelihood depends strongly on the number of mutation steps, and it is not obvious how to determine these a priori. The commonly used potential scale reduction factor $\hat{R}$ can be used to determine convergence of the approximation of the posterior (Gelman & Rubin, 1992), but this does not seem to imply any bounds on the marginal likelihood estimate. To illustrate this, recall Fig. 6, which shows the marginal likelihood as a functional of the number of mutations. At $S=2\,560$, $\hat{R}<1.1$, commonly used as a threshold for convergence, but the marginal likelihood estimate is still far from the bridge sampling estimate. The intricate relationship between the mutation phase of SMC and the adaptive-tempering procedure, and their impact on the marginal likelihood estimate, is not completely clear, although recent work has set important steps in this direction (Beskos et al., 2014; Dai et al., 2022). Of course, this remains a fundamentally challenging problem, in particular for high-dimensional models, that also affects other approaches such as bridge sampling (Gronau et al., 2017) or nested importance sampling (Tran et al., 2021). These have both proven to be effective, but have drawbacks as well; for instance, Wong et al. (2020) point out that particular steps within the bridge sampling approach can introduce a bias to the estimator. For SMC, Chopin and Papaspiliopoulos (2020) show that the tempered variant of this algorithm results in an unbiased estimate, but also indicate that such a guarantee does not exist when the algorithm is adaptive. Lastly, sampling until convergence is a common process with MCMC, with several diagnostic heuristics available that help the practitioner. With MCMC one can simply proceed to sample for longer until convergence is reached, but with the adaptive-tempered SMC algorithm the entire procedure is restarted from scratch. This can make the process of finding the right number of mutation steps wasteful. Recent work by Dau and Chopin (2022) suggests how this waste can be avoided, but further developments are needed to streamline the procedure.

Despite these drawbacks, SMC appears to be a highly promising tool for computational psychology (Dai et al., 2022). The SMC procedure can be improved further to be (even) more efficient, both for approximating posterior distributions, as well as for estimating marginal likelihoods. For example, Buchholz et al. (2021) and Salomone et al. (2023) discuss the use of Hamiltonian Monte Carlo (HMC) for the mutation steps, rather than Metropolis–Hastings, and also investigate how the parameters of HMC can be adapted based on previous SMC iterations. This makes exploring each tempered distribution more efficient, potentially greatly reducing the number of mutation steps needed at every iteration. Even more straightforward is to re-calibrate the proposal distribution of the mutation step in between the different tempered distributions (Dau & Chopin, 2022).

In the future, the uptake of SMC as a tool for inference and model comparison will depend critically on its availability in easy-to-use probabilistic programming languages (PPLs). Currently, the de facto standard for such software is the (adaptive) NUTS algorithm, which is available in most of the popular languages such as Stan (Carpenter et al., 2017). To the best of our knowledge, only PyMC (Oriol et al., 2023) currently offers an (adaptive and tempered) SMC implementation. For our examples, we made use of the Blackjax (Cabezas et al., 2023) Python library. On top of this library, we have developed the Bayesian modeling toolbox bamojax to streamline the modeling and implementation process. We hope that this toolbox lowers the threshold for Bayesian practitioners to try out SMC, and others may experiment and adopt SMC in their own work. We provide all code for the examples in this paper at our GitHub repository.

## Data Availability

The applications cover data sets that were published previously. For the variable selection data, a description of the data as well as the procedure to acquire them is provided here. The student GPA data is described at the JASP website. Lastly, the data used for the Iowa Gambling Task (see Gronau et al., 2017) are available at the Open Science Framework.

## A Variable selection in linear regression

### A.1 The prior

As discussed in Section “Inference: Variable selection in depression”, we implement the continuous analogue of the spike-and-slab distribution suggested by Thomson et al. (2019) as the prior on the regression coefficients. For comparison, we also show the traditional spike-and-slab distribution here George and McCulloch (1993):

22 $$\begin{aligned} z_j&\sim \text {Bernoulli}(0.5) &  j=1, \ldots , p\\ \tau&\sim \text {InverseGamma}(0.05, 0.05) &  \\ \beta _j&\sim z_j\mathcal {N}\left( 0, \tau ^2 \right) + (1-z_j) \delta _0 &  j=1, \ldots , p , \end{aligned}$$

where $\delta _0$ represents the Dirac delta, which can loosely be interpreted as a distribution with density zero everywhere except at 0, and that integrates to 1 as a probability density function:

23 $$\begin{aligned} \int _{-\infty }^{\infty } \delta _0(x) {\text {d}}\!{x} = 1 . \end{aligned}$$

For variable selection, $\delta _0$ is known as the *spike*, and $\mathcal {N}(0, \tau ^2)$ as the *slab*. However, since the spike-and-slab distribution contains the discrete latent parameters $z_j$, it cannot be used in conjunction with gradient-based MCMC algorithms such as NUTS. As an alternative, Thomson et al. (2019) introduced a continuous generalization:

24 $$\begin{aligned}\text {logit}(\lambda _j)&\sim \mathcal {N}\left( \mu _\lambda , \sigma _\lambda \right) &  j=1, \ldots , p\\ \tau&\sim \text {InverseGamma}(0.05, 0.05) &  \\ \beta _j&\sim \mathcal {N}\left( 0, (\lambda _j \tau )^2 \right) &  j=1, \ldots , p . \end{aligned}$$

We set $\mu _\lambda = \text {logit}(0.2)$ and $\sigma _\lambda =1.0$. In comparison to the spike-and-slab distribution above, $\lambda _j$ plays the role of a continuous extension of the selector variable $z_j$; if $\lambda _j \rightarrow 0$, the variance of the distribution of $\beta _j$ is reduced to zero, mimicking the Dirac delta function. If instead $\lambda _j\rightarrow 1$, the distribution on $\beta _j$ is simply a Gaussian with width $\tau$, similar to the slab distribution.

### A.2 Inference settings

For all three approaches, that is, MH-MCMC, Gibbs-MCMC, and NUTS HMC, as well as MH-in-SMC, Gibbs-in-SMC, and NUTS-in-SMC, we increased the number of samples or mutation steps until convergence was attained, as determined by a PSRF (see Section “Evaluating the posterior”) score below 1.1 for all model parameters.

For MH, we used Gaussian proposals with a step size of $\sigma =0.001$. For Gibbs, Gaussian proposals where used for each variable, with step sizes of 0.001, 0.01, 0.01, and 0.2 for the variables $\beta$, $\lambda$, $\sigma$, and $\tau$, respectively. For NUTS, we used window adaptation for 500 samples to determine the optimal step size and inverse mass matrix adaptively. All MCMC output was downsampled to 1000 samples after convergence.

The adaptive-tempered SMC algorithm used $M=1000$ particles and the fraction of desired effective samples was set to $\alpha =0.5$. The mutation kernels used the same step sizes as in the MCMC approach.

### A.3 List of variable names

**Table 2:** Variables used in the linear regression variable selection experiment

Abbreviation	Full variable name
–	Age
–	Gender
Nicotine	Nicotine dependence
ATQ 82	Negative Affect
ATQ 90	Extraversion/Surgency
ATQ 94	Orienting Sensitivity
ATQ 86	Effortful Control
IPRI 29	Perspective-Taking Scale
IPRI 30	Fantasy Scale
IPRI 31	Empathic Concern Scale
IPRI 32	Personal Distress Scale
DOSP 91	Ethical Subscale (Risk Taking)
DOSP 94	Recreational Subscale (Risk Taking)
DOSP 95	Social Subscale(Risk Taking)
DOSP 92	Financial Subscale (Risk Taking)
DOSP 93	Health/Safety Subscale (Risk Taking)
UPPS 60	Negative Urgency Score
UPPS 61	Premeditation Score
UPPS 62	Perseverance Score
UPPS 63	Sensation Seeking Score
UPPS 64	Positive Urgency Score
PSQI 18	Total Sleep Disturbance
IPAQ 72	Total Physical Activity Met

Table 2 lists the variables that were used in the variable selection experiment in Section “Inference: Variable selection in depression”.

## B The growth mixture model

### B.1 Priors for the GrMM

The growth mixture model (GrMM) as discussed in Section “Multimodality: A growth mixture model for grade point averages” is completed by defining the following prior distributions for its latent parameters:

25 $$\begin{aligned}  \beta _{dk}\sim \mathcal {N}(0, 2) &  \qquad d=0, \ldots , D, \qquad j=2, \ldots , K\\ \tilde{w}_1= 0 &  \\ \tilde{w}_k\sim \mathcal {N}(0, 1) &  \qquad k=2, \ldots , K \\ \textbf{w}= \text {softmax}\left( \tilde{\textbf{w}}\right) &  \\ \log \sigma _k\sim \mathcal {N}(0, 1) &  \qquad k=1, \ldots , K,  \end{aligned}$$

in which

26 $$\begin{aligned} \text {softmax}(\textbf{w})_k = \frac{\exp (w_k)}{\sum _{k'=1}^K \exp (w_{k'})} . \end{aligned}$$

The wider prior on the regression coefficients $\beta _{dk}$ ensures that the SMC algorithm has a wide variety of initial particles. The centered softmax transformation for $\textbf{w}$, instead of, for example, a Dirichlet distribution, is a pragmatic choice. It allows us to easily create proposals $\textbf{w}^* \sim q(\textbf{w}^* \mid \textbf{w})$; we simply take Gaussian steps for each of the elements $\tilde{w}_k$ (with $k=2, \ldots , K)$ and using the softmax transformation we ensure that $\textbf{w}$ is a vector of probabilities.

### B.2 Inference settings

Since in the growth mixture model example, the PSRF measure does not provide a useful indicator of convergence (due to the presence of multiple modes), we instead sampled until the posterior expectation of the log-likelihood was within 2% of a long NUTS run. For MCMC, this required 2, 000, 000 samples for MH, $1\,250\,000$ for Gibbs, and 20, 000 for NUTS. All MCMC output was then downsampled to 2000 samples. For SMC with $M=2000$ particles, the same approach resulted in 800 mutation steps for MH, 50 for Gibbs, and 1 for NUTS.

For MH, we used a Gaussian proposal distribution with a step size of 0.01. For Gibbs, Gaussian proposals were used for each variable, all with a step size of 0.1. For NUTS, window adaptation for 500 samples was used to determine the step size and inverse mass matrix. The same settings were used for MCMC and for using these MCMC kernels within SMC.

### B.3 Quantifying multimodality

To determine how robust the inference methods are when dealing with multimodal posterior distributions, we make use of the fact that in the growth mixture model the component labels can be permuted arbitrarily. This means that if the multimodality is captured well, the respective distribution of the slope and intercept parameters $\beta _{0k}$ and $\beta _{1k}$ should be the same across the different components $k\in 1, \ldots , K$; all of the modes should be represented in each. This further means that, for example, $p(\beta _{0k} \mid \textbf{x}, \textbf{Y})$ and $p(\beta _{0j} \mid \textbf{x}, \textbf{Y})$ should be the same, for $k\ne j$. We quantify this similarity using the Kullback–Leibler divergence:

27 $$\begin{aligned} KL(p\Vert q) \approx \sum _{x\in \mathcal {X}} p(x) \log \frac{p(x)}{q(x)} , \end{aligned}$$

where $x\in \mathcal {X}$ indicates we sum over a linearly spaced grid of the domain of the input variable, here $\beta _{0k}$ and $\beta _{1k}$, respectively. In our implementation, *p* and *q* are derived using a Gaussian kernel density estimator applied to the samples/particles of the approximate inference schemes, which results in smooth densities that can be queried at every *x*. Furthermore, to avoid division-by-zero errors, a small number is added to both *p*(*x*) and *q*(*x*).

## C The expectancy-valence model

### C.1 Priors for the EV model

The following uninformative priors were used for the priors in the original expectancy-valence model::

28 $$\begin{aligned} {\begin{matrix} w & \sim \text {beta}(1.0, 1.0) \\ a & \sim \text {beta}(1.0, 1.0) \\ \hat{c} & \sim \text {beta}(1.0, 1.0) \\ c & = 4\hat{c} - 2 , \end{matrix}} \end{aligned}$$

in which the last step ensures that $c \in [-2, 2]$, as desired. For the hierarchical extension, the following prior structure is used (Wetzels et al., 2010; Gronau et al., 2017):

29 $$\begin{aligned} {\begin{matrix} \mu _w & \sim \mathcal {N}(0.0, 1.0)\\ \mu _a & \sim \mathcal {N}(0.0, 1.0)\\ \mu _c & \sim \mathcal {N}(0.0, 1.0)\\ \\ \hat{w}_i & \sim \mathcal {N}(\mu _w, \sigma _w) \\ \hat{a}_i & \sim \mathcal {N}(\mu _a, \sigma _a) \\ \hat{c}_i & \sim \mathcal {N}(\mu _c, \sigma _c) \\ \end{matrix}}\qquad \qquad {\begin{matrix} \sigma _w & \sim \mathcal {U}(0.0, 1.5)\\ \sigma _a & \sim \mathcal {U}(0.0, 1.5)\\ \sigma _c & \sim \mathcal {U}(0.0, 1.5)\\ \\ w_i & = \Phi (\hat{w}_i) \\ a_i & = \Phi (\hat{w}_i) \\ c_i & = 4\Phi (\hat{w}_i)-2 , \end{matrix}} \end{aligned}$$

where $i=1, \ldots , N$, and $\Phi (\cdot )$ is the standard Gaussian cumulative density function, which ensures that the parameters have the correct support.

### C.2 Inference settings

#### C.2.1 Independent EV model

For Metropolis–Hastings within SMC we used a Gaussian proposal distribution with a step size of $\sigma =0.01$, and $S=100$ mutations. For Gibbs within SMC, for each of the variables *w*, *a*, and *c* a Gaussian proposal distribution was used with a step size of $\sigma =0.05$, and $S=50$ mutations. For NUTS HMC, we used two warm-up samples to determine the inverse mass matrix and step size adaptively, and $S=20$ mutations. Computation of the log marginal likelihoods for all 30 subjects took about 1 min for all three methods.

#### C.2.2 Hierarchical EV model

For MH, we used a step size of $\sigma =0.01$. We first set the number of mutations to $S=500$, and then doubled it if the computation time per chain was smaller than the computation time for the baseline (see below). The final MH-in-SMC run used $S=64,000$ mutation steps and took 14 h.

For Gibbs, we used Gaussian proposals for each of the individual variables of the hierarchical EV model. For parameters $\mu _w$, $\mu _a$, and $\mu _c$, we set the step size to $\sigma =0.5$, for $\sigma _w$, $\sigma _a$, and $\sigma _c$ this was set to $\sigma =0.2$, and finally for *w*, *a*, and *c* the step size was $\sigma =0.02$. The first run used $S=100$ mutation steps, whereas the final run used $S=25\,600$ steps and took 18 h to finish.

For NUTS, we again used window adaptation to determine the ideal step size and inverse mass matrix. However, this analysis took exceedingly long (while returning log marginal likelihoods still far below the baseline) so that this approach was aborted. We conclude that NUTS-in-SMC is too slow for high-dimensional models like this.

### C.3 Bridge sampling

The bridge sampling approach by Gronau et al. (2017) consists of two steps. In the first, the posterior distribution of the hierarchical EV model is approximated using MCMC, as implemented in the JAGS software (Plummer, 2003). Two MCMC chains of 150,000 samples are sampled, of which the first 30,000 are discarded to allow the sampler to reach the target distribution. The remaining 120,000 samples per chain are then reduced to 30,000 by keeping every $4^{\text {th}}$ sample and discarding the rest. In the second step, the first half of the samples is used to obtain a tailored proposal distribution. The second half of the samples is then used in an iterative scheme to estimate the marginal likelihood (for the details of bridge sampling we refer to Gronau et al. (2017)). Finally, the entire procedure is repeated ten times to obtain an estimate of the reliability of the approach. Per run, this required approximately 9 h.

## Footnotes

1 When the prior $p(\theta )$ is conjugate to the likelihood $p(D\mid \theta )$; In this case, by construction, the posterior will be of the same distribution class as the prior, but with updated parameters. This is convenient and efficient, but rare in non-trivial Bayesian models.

2 In practice, of course, one works with log-densities and hence log-weights, for numerical stability.

3 For more details on these estimators, as well as a state-of-the-art approach to estimating the marginal likelihood, we refer the reader to Gronau et al. (2017), as well as to Fourment et al. (2020) and Llorente et al. (2023) for recent reviews.

4 One might be surprised to see the product in Eq. (11) start at $t=2$. However, recall that $\Delta \beta _t = \beta _t - \beta _{t-1}$, which is not defined for $t=1$. Instead, for the first iteration, the temperature is simply 0. At this temperature, and with uniformly initialized weights, the contribution to the marginal likelihood is simply a multiplication with 1.0. A more detailed explanation is provided in (Chopin and Papaspiliopoulos, 2020, Eq. (16.9)).

## Code Availability

The Python code for all examples in this manuscript are available at our GitHub repository.
